# Web-Based Dietary and Physical Activity Intervention Programs for Patients With Hypertension: Scoping Review

**DOI:** 10.2196/22465

**Published:** 2021-03-15

**Authors:** Fatimah Najihah Baderol Allam, Mohd Ramadan Ab Hamid, Siti Sabariah Buhari, Harrinni Md Noor

**Affiliations:** 1 Centre for Dietetics Studies Faculty of Health Sciences Universiti Teknologi MARA Puncak Alam Malaysia; 2 Faculty of Education Universiti Teknologi MARA Puncak Alam Malaysia

**Keywords:** hypertension, blood pressure, education, website-based, dietary intake, physical activity

## Abstract

**Background:**

Hypertension is the root cause of many chronic diseases. Lifestyle changes (ie, dietary alterations and physical activity) are seen to be an important step in the prevention and treatment of hypertension. Educating people through web-based interventional programs could offer an effective solution and help these patients with hypertension in the existing health care scenario.

**Objective:**

In this study, the researchers conducted a scoping literature review of the web-based dietary changes and physical activity–related intervention programs designed for the patients with hypertension and identified the methodologies, effectiveness, protocols, and theories, which could affect and improve existing clinical activities.

**Methods:**

This review followed the scoping review methodology to identify and process the peer-reviewed studies published between 2010 and 2020. The literature searches were conducted on the following electronic databases: PubMed, Web of Science, MEDLINE (Medical Literature Analysis and Retrieval System Online), ScienceDirect, Scopus, and Google Scholar. By using relevant search terms, studies were included if they offered information related to the web-based intervention tools, specifically dietary and physical activity intervention for patients with hypertension. Studies written or translated in English language and published within the date range (January 2010 to March 2020) were included.

**Results:**

Overall, 1441 articles were initially identified. The reviewers included 35 articles after removing duplicates and screening titles. Only 21 articles were assessed for full review, and 15 were kept for analysis. The researchers selected 15 web-based intervention articles published on the topic of hypertension from 7 countries. A few of these 15 web-based tools (4, 27%) included more than 3 functions and provided a lot of important information (such as appointments, health records, or viewable care). Several tools were standalone tools (11, 73%), while most of the tools supported communication intervention–related lifestyle or behavioral changes (13, 87%) and medication adherence (6, 40%). It was found that physicians (9, 60%), allied health professionals (5, 33%), and nurses (5, 33%) were the health care providers who generally used these tools for communicating with their patients. More than half of the above tools (10, 67%) were assessed by different researchers in randomized controlled trials, while 5 tools (33%) were investigated in nonrandomized studies.

**Conclusions:**

We identified many web-based intervention programs for patients with hypertension from the literature databases. The findings indicate that numerous benefits can be derived after using a web-based dietary and physical activity intervention program for hypertension focusing on lifestyle changes. However, developers need to consider the preferences of the patients with regard to the information or the design features while developing or modifying web-based educational websites. These tools could be used for designing a patient-tailored website intervention program that is based on diet and physical activities for patients with hypertension.

## Introduction

### Background

Hypertension, or high blood pressure, is the root cause of several chronic diseases and affected more than 31.1% of the adults (1.39 billion) around the world in 2010 [1]. One study stated that in the last 20 years, the high-income countries showed a slight decrease in the prevalence of high blood pressure, whereas the low- and middle-income countries (LMICs) showed a significantly higher prevalence of the disease. These differences in the trend have increased the burden of hypertension and other related cardiovascular issues on the health care systems of these LMICs [2].

The prevalence of this health issue has increased around the globe due to many lifestyle changes like an unhealthy diet (which is low in potassium and high in sodium) and a lack of physical activity. Furthermore, many factors such as occupation, abdominal obesity, body mass index, education levels, tobacco and alcohol usage, and the socioeconomic status of the people also contributed to hypertension [2,3]. An increased prevalence of high blood pressure is also responsible for other major diseases like diabetes, cardiovascular problems, and chronic kidney disease [4-6]. Fortunately, blood pressure can be controlled by improving lifestyle changes through the emergence of educational programs [7].

### The Effect of Web-Based Educational Interventions in Lifestyle Modification

In a previous study, Ali et al stated that educational programs are an effective technique that can be used by patients with hypertension to increase their knowledge about lifestyle changes, controlling their harmful lifestyle habits, and enhancing their self-management [7]. They observed that after using these tools for 3 months, there was a significant decrease in the number of excessive-salt consumers, physically inactive people, and those who consumed an inadequate amount of fish and fiber. This indicated that the tools helped in improving the lifestyle scores of the patients. By engaging with educational intervention as one approach, this can be effective in promoting physical activity and improving weight management, nutrition, and mental health in patients with hypertension [8].

Since there is an increase in the global prevalence of hypertension, people need to undergo an urgent transformation. In the past few years, there has been an increase in many digital health-related interventions, such as mobile apps and web-based and software-based interventions. These tools allow the patients to access all health-related information and encourage them to follow lifestyle and behavioral changes [9,10]. Web-based educational interventions are suitable to be used as a medium to promote the exchange of patient education information through online mechanisms such as websites [11]. This medium of interventions allows for flexibility in the type and amount of information people can access on a routine basis [12]. This type of web-based intervention also can encourage patients to manage their high blood pressure by changing their lifestyle habits.

### Objective

To the best of our knowledge, previous literature reviews have not incorporated a comprehensive list of studies on web-based dietary and physical activity–related intervention programs for patients with hypertension. Therefore, the objective of this scoping review was to provide a comprehensive view of the literature on the use of web-based dietary and physical activity–related intervention programs for patients with hypertension. This review sought to identify and summarize the methodologies, effectiveness, protocols, and theories, which enables us to have a better understanding of the web-based intervention programs that could influence patients’ eventual health outcomes and improve existing clinical operations.

The researchers have carried out a scoping review of the studies published in the literature that were related to the digital and web-based dietary or physical activity intervention programs targeted for patients with hypertension. This review was based on the 5-point framework that was proposed by Arksey and O’Malley [13] and attempted to answer some questions as follows: (1) What were the focus areas or characteristics of the web-based learning programs? (2) What studies or interventions were based on the web-based intervention programs for managing hypertension? (3) What were the measurement outcomes for the web-based learning programs? (4) What was the distribution of studies that were focused on web-based intervention programs for supporting hypertensive disorders in the past 10 years?

## Methods

### Scoping Review Methodology

In this study, a scoping review methodology is chosen to acquire an overview of the extant literature on web-based dietary and physical activity–related intervention programs for patients with hypertension. A scoping review is a form of literature review that is useful for mapping applicable literature in a field of interest. The researchers carried out a scoping review based on the 5-step framework proposed by Arksey and O’Malley: (1) identifying the research objectives and search strategies; (2) identifying the relevant research studies; (3) study selection; (4) extracting and charting the data; and (5) collating, summarizing, and reporting all results [13].

A scoping review technique is very helpful for reviews that determine the range, extent, or nature of a specific topic. It is also used for classifying the main elements in a field or for identifying the gaps that exist in the related literature [14]. This review approach is also helpful if the study area is very broad or there is little or no information available about the topic. In such situations, a formal systematic review (which focuses on the study design and includes limiting selection criteria) limits the amount of information that can be acquired.

This review was conducted following the guidelines of the PRISMA-ScR (Preferred Reporting Items for Systematic reviews and Meta-Analyses extension for scoping review) [15]. PRISMA refers to the evidence-based minimal set of items to be reported for systematic reviews or meta-analyses. It includes a 27-item checklist along with a 4-phase flow diagram that helps all researchers report the retrieved data. The purpose of the PRISMA-ScR is to help readers develop a greater understanding of relevant terminology, core concepts, and key items to report for scoping review. Furthermore, it helps the researchers critically appraise all the previously published systematic reviews [16].

### Search Strategy

The researchers carried out a search for the various web-based dietary and physical activity–related intervention programs for patients with hypertension in May 2020, with the help of a few comprehensive search databases. Table 1 presents the list of search terms that were used by the researchers. Since the objective of this study was to determine the web-based tools used in the health care sector, the researchers have focused their complete search on a few specific websites, such as PubMed, Web of Science, MEDLINE (Medical Literature Analysis and Retrieval System Online), ScienceDirect, Scopus, and Google Scholar. They used a combination of keywords, which included terms like web-based, diet, and physical activity. They set the search string with the help of an iterative process for including all the related keywords (Multimedia Appendix 1). They also studied the bibliography and the list of references included in the articles to determine the relevant studies. Furthermore, they manually searched for the keywords so that they could identify all the articles that they missed in the reference lists or databases.

**Table 1:** The search terms that were used by the researchers.

Keywords	Synonymous terms
Web-based	Mobile health OR internet OR mhealth OR digital OR eHealth OR web application OR internet-enabled interactive multimedia OR internet-supported online OR technology-assisted OR computer-assisted OR website-delivered OR computer-based OR computer-delivered OR internet-based OR interactive computer-based OR e-learning OR website OR digital medicines OR digital health technology
Nutrition intervention	Diet intervention
Physical activity	Exercises
Hypertension	Hypertensive OR high blood pressure

### Selection Criteria

The researchers screened for the articles that were to be included in 2 stages: (1) title and abstract reviews and (2) complete article review. They used the scoping review technique for this purpose.

### Eligibility and Exclusion Criteria

The aim was to include articles that describe web-based dietary and physical activity–related intervention programs for patients with hypertension. Studies were included if they (1) were an original paper published in a peer-reviewed journal (except review papers); (2) included website-based interventions for hypertension; (3) included diet, physical activity, or both as part of the intervention in the web-based intervention tools; (4) reported outcomes from an intervention using a website or digital health solution that involved use of the web for the same domain; (5) had adults diagnosed with hypertension as participants; (6) were published between January 2010 and March 2020; and (7) were written or translated in the English language.

The exclusion criteria were as follows: (1) studies targeting very specific groups of individuals (pregnant women, children, or athletes), (2) interventions only targeting health care providers, (3) studies written in other languages or not translated in English language, and (4) studies published in research avenues other than research journals. However, studies targeting people who are obese or diabetic were included as these diseases are directly linked to dietary habits and more prevalent in the general population.

### Study Identification

The primary author (FNBA) carried out a literature search with the help of a search string (Multimedia Appendix 1). They screened the retrieved titles and the abstracts to eliminate the duplicate search results. They also eliminated all articles that were not related to the web-based dietary or physical activity–based intervention program.

### Article Selection

Thereafter, 3 independent reviewers including the primary author (FNBA, MRAH, and SSB) reviewed the results and filtered the potentially related articles and the articles that were labelled as *maybe* by the primary author by using the above-mentioned inclusion and exclusion criteria. All discrepancies were resolved by consensus, and the researchers calculated the Cohen κ coefficient for measuring the interrater agreement (κ=0.76), which indicated a substantial agreement. Finally, the researchers acquired the full-text articles from their filtered list. Thereafter, 2 authors (FNBA and MRAH) reviewed all the acquired full-text articles and eliminated the articles that did not fulfil the inclusion and exclusion criteria.

### Data Extraction

All data were extracted from the articles that were selected. These data have been described based on the (1) article characteristics (ie, which disease the study was based on and the study settings); (2) tool characteristics, that is, structure (medium of communication), function (additional features such as the viewable care plan), and communication paradigm (ie, one-to-many or one-one communication flow); (3) intended usage, context, and users included in the study; and (4) evaluation of the study (ie, design, evaluation stages, and outcomes).

## Results

### Included Studies

After searching for relevant studies in the literature, the researchers retrieved 1441 studies from the 6 databases used: ScienceDirect (n=814), Scopus (n=206), PubMed (n=178), Web of Science (n=114), MEDLINE (n=107), and Google Scholar (n=22). After duplicates were eliminated, 949 results were obtained, out of which 914 studies were excluded based on their titles after the screening process.

**Figure 1:**
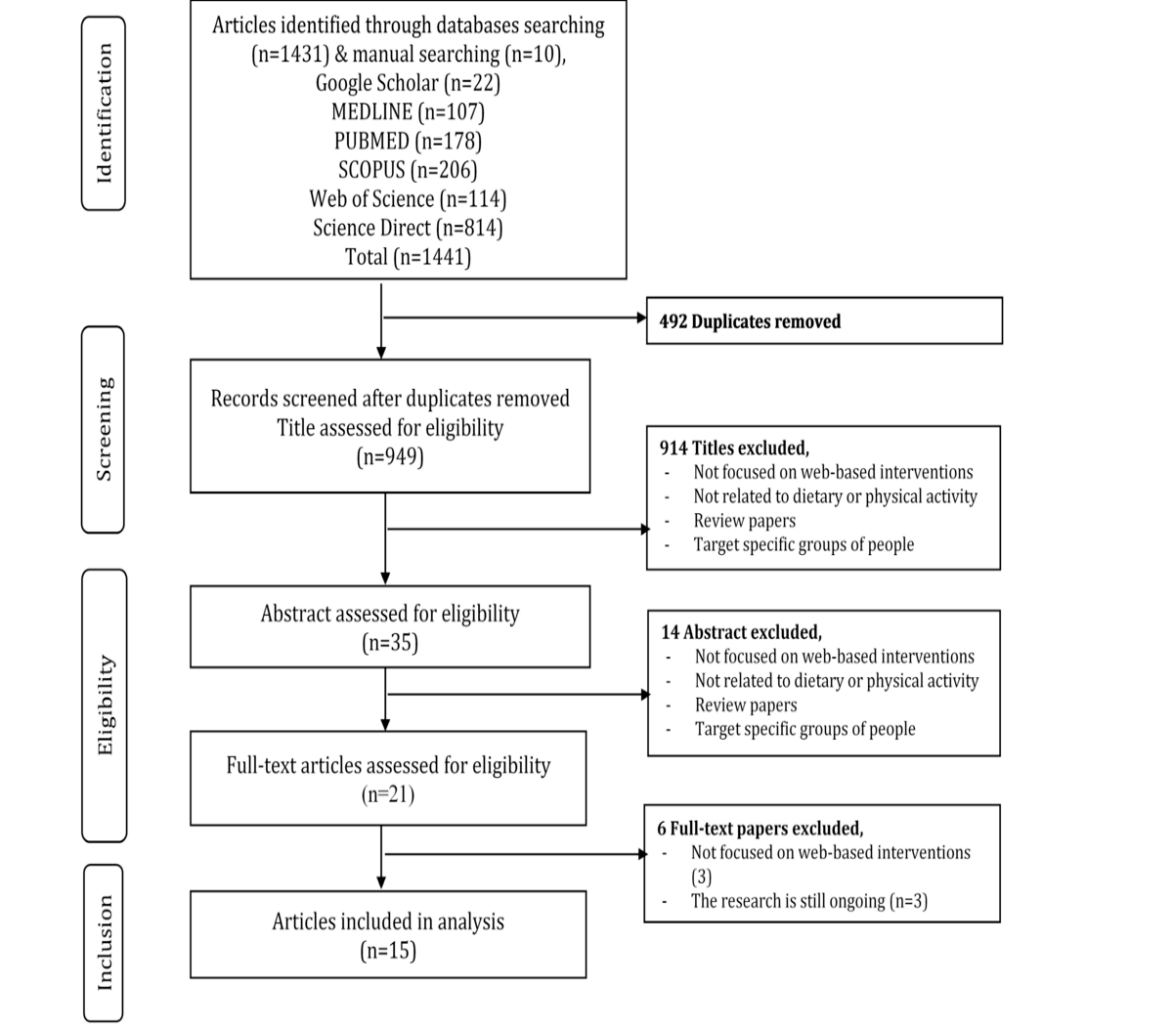
Flow diagram depicting the inclusion of studies that used web-based dietary and physical activity intervention programs for patients with hypertension.

Thereafter, the researchers screened the remaining 35 studies based on their titles and abstracts and downloaded 21 full-text articles. These articles were further reviewed based on the inclusion and exclusion criteria, which finally led to 15 articles (Figure 1). The 6 articles were excluded either because they did not include a web-based tool (n=3) or because research was still in progress (n=3). Lastly, the researchers extracted the data from the final 15 articles.

### Description of the Web-Based Tools Included in the Study

#### Article Characteristics

The earliest article that was published in the past decade was in 2011. Since then there was a significant increase in the number of studies that were published between 2011 and 2019, except in 2013 (Table 2). Of the 15 studies, most were conducted in the United States (5, 33%) and Canada (4, 27%) (Table 2). Many of these studies were carried out at primary care centers (11, 73%), which included the target adult population (15/15, 100%).

#### Characteristics of the Web-Based Tools

The web-based tools were characterized by their tool structures, functions, and communication paradigms. Table 3 presents these characteristics.

**Table 2:** Characteristics of the published articles (n=15).

Characteristics		n (%)
**Publication year (N=15)**		
	2011	1 (7)
	2012	2 (13)
	2013	0 (0)
	2014	1 (7)
	2015	1 (7)
	2016	2 (13)
	2017	2 (13)
	2018	3 (20)
	2019	3 (20)
	2020	0 (0)
**Publication country of origin (N=15)**		
	England	1 (7)
	United States	5 (33)
	Canada	4 (27)
	Spain	1 (7)
	Korea	2 (13)
	Italy	1 (7)
	Taiwan	1 (7)
**Unique study type (N=15)**		
	Original study	12 (80)
	Protocol	1 (7)
	Other: pilot study	2 (13)
**Study context or setting of use (N=15)**		
	Primary care	11 (73)
	Tertiary care outpatient clinics	1 (7)
	Integrated health care organization	1 (7)
	Workplace	1 (7)
	Unknown	1 (7)
**Population (N=15)**		
	Adult	15 (100)
**Disease or clinical area of interest (N=18)^a^**		
	Cardiovascular disease	2 (11)
	Hypertension	12 (67)
	Metabolic syndrome	1 (6)
	Diabetes	1 (6)
	Hyperlipidemia	1 (6)
	Obesity	1 (6)

^a^A few of the above studies assessed the tools in several contexts, for instance, to assess both mental health and diabetes in the patients.

**Table 3:** Tool characteristics, intended use, and users (n=15). The table classifies variables according to unique tools rather than individual studies as the unit of analysis.

Characteristics			n (%)
**Structures**			
	**Medium of communication or format^a^**		
		Website-based	15 (100)
		Software-based connected by the internet	1 (7)
		Telephone calls	1 (7)
		Native application	2 (13)
	**Component of another platform (n=15)**		
		Patient portal	1 (7)
		Electronic health record	2 (13)
		Digital management program	1 (7)
		Standalone	11 (73)
**Functions**			
	**Communication structure (n=15)**		
		One-way communication	5 (33)
		Two-way communication	10 (67)
	**Type of communication (n=15)**		
		Unstructured communication (patient-provider free-form dialogue)	8 (53)
		Structured communication (tailored feedback)	7 (47)
	**Tools with functions beyond patient-provider communication component^a^**		
		With 3 or more additional functions	4 (27)
		Linked to a health record	3 (20)
		Linked to appointment or scheduling	2 (13)
		Linked to viewable care or treatment plan	2 (13)
		Linked to prescription renewal	1 (7)
		Linked to laboratory or test results	1 (7)
		Linked to symptom diary or tracker	1 (7)
		Linked to disease information or education	13 (87)
		Linked to consultations	2 (13)
**Communication paradigm**			
	Asynchronous		9 (60)
	Of asynchronous tools, time-limited (response from a provider within a specified time window)		1 (7)
	Synchronous		5 (33)
	Both		1 (7)
**Intended use and users**			
	**Intended use of communication intervention^a^**		
		Lifestyle or behavior modification	13 (87)
		Symptom reporting	1 (7)
		Care planning	3 (20)
		Medication adherence	6 (40)
		Not specified	1 (7)
	**Type of health care provider intended to use tool with patients or caregivers^a^**		
		Nurse	5 (33)
		Physician	9 (60)
		Pharmacist	2 (13)
		Allied health professional	5 (33)
		Health advisor	1 (7)
		Not specified	1 (7)
**Other**			
	**Compensation to health care provider (n=15)**		
		Did not provide compensation	14 (93)
		Did provide compensation	1 (7)
	**Tool access (n=15)**		
		Free through research participation	13 (87)
		Prior registration required via website or service	2 (13)
	**URL available in article (n=15)**		
		Yes	4 (27)
		No	11 (73)

^a^A few of the studies used multiple variables. For instance, with regard to the communication medium, one study used web-based tools and telephone calls.

##### Structures

A majority of the selected 15 web-based tools had a 2-way communication system between the patients and the providers (10, 67%). They used the website as the mode of communication. However, a few of these tools used multiple modes of communication, such as software-based systems that were connected by internet services (1, 7%), telephone calls (1, 7%), or native applications (2, 13%), which were developed as computer software or a smartphone app.

All the above-mentioned web-based tools were designed as educational or informational websites. However, only some of these web-based tools belonged to a different platform having multiple functions and features such as electronic health records (2, 13%), digital management programs (1, 7%), or a patient portal (1, 7%); 73% (11/15) of the tools were standalone communication tools.

##### Functions

The researchers identified 2 types of communication functions (ie, unstructured and structured communication). Many of these tools (8, 53%) included an unstructured communication system that allowed the providers and patients to engage in a free-form dialogue. Of the 15 web-based tools, 7 included a structured communication type, wherein the patients submitted their queries, which generated a response from their providers.

Furthermore, 87% of these tools (13/15) provided disease-related information and educated the people. In addition, a few of these tools offered different functions, that is, they were linked to the health records of the patients (3, 20%), appointments or schedules (2, 13%), treatment plans and a viewable care plan (2, 13%), consultation (2, 13%), prescription renewals (1, 7%), test or laboratory results (1, 7%), or a symptom tracker or diary (1, 7%).

##### Communication Paradigm

Most of these web-based tools (9, 60%) used an asynchronous communication system. Out of the different asynchronous tools, a single web-based tool showed a time limitation (7%). However, 5 of the 15 web-based tools used a synchronous communication system (33%), while 1 tool used both the communication types (7%).

##### Intended Use, Context, and Users

Based on the intended use of the various web-based tools, they were categorized into 4 different classes: behavior or lifestyle modifications (13, 87%), adherence to medicines (6, 40%), care planning (3, 20%), and reporting of symptoms (1, 7%). Out of the 15 web-based tools, 1 did not specify its intended use in the article, since the website only assessed the number of visitors who returned to the website.

A majority of the selected studies were conducted in a single critical field of study, that is, hypertension (12/15, 80%), while some papers used these web-based tools to evaluate multiple types of diseases such as cardiovascular diseases (2/18, 11%), diabetes (1/18, 6%), hyperlipidemia (1/18, 6%), metabolic syndromes (1/18, 6%), and obesity (1/18, 6%).

The health care workers who mainly used the web-based tools were physicians (9, 60%). In 5 of the selected studies, nurses and other allied health professionals like dietitians (33%) also used these tools. Of these studies, 13% included pharmacists (2/15), while 7% included health advisors. One study did not specify the health care provider who used their tool. Out of the 15 selected studies, 1 study stated that the participants were offered monetary compensation for their participation.

#### Evaluation Characteristics

##### Study Design and Study Stage

Table 4 presents the evaluation of the characteristics displayed by the selected studies. 10 of the selected studies were randomized controlled trials (RCTs), while the remaining 5 were nonrandomized studies. Out of these nonrandomized studies, 2 included a quasi-experimental pretest versus posttest design, while 2 others were pilot studies and 1 included a website analysis. With regard to the stage of the study, based on the 2008 Medical Research Council Framework for Evaluation of Complex Interventions, 87% of the selected studies (13/15) were in their evaluation phase (10 RCTs and 3 nonrandomized studies). The remaining 2 nonrandomized studies were in their feasibility and piloting phases (13%).

The sample sizes of the RCT studies ranged between 106 and 9298 patients. These studies spanned for 4-12 months for follow-up. On the other hand, the sample sizes of the nonrandomized studies ranged between 56 and 803 patients for the quasi-experimental pretest versus posttest study design, while they ranged between 51 and 690 patients for the pilot studies.

**Table 4:** Assessment of the characteristics of unique studies (n=15). Unique studies were defined as the studies that led to several publications. These did not include editorials, protocols, or commentaries.

Study design and evaluation characteristics				Value
**Randomized controlled trials (n=10)**				
	**Communication component feature type, n**			
		Primary feature	5	
		Supplemental feature	5	
	Stage of study^a^		Evaluation	
	**Types of results captured in each study^b^, n**			
		Acceptability	2	
		Clinical	8	
		Psychological	1	
		Behavioral	5	
		Usage	1	
		Willingness	1	
		Cost-effectiveness	1	
	Sample size, median (IQR; range)		264 (165.75-369.25; 106-9298)	
	Length of follow-up (months), median (IQR; range)		12 (6.75-12; 4-12)	
Nonrandomized studies (n=5)				N/A^a^
**Quasi-experimental pretest vs posttest design (n=2)**				
	**Communication component feature type, n**			
		Primary feature	1	
		Supplemental feature	1	
	Stage of study^a^		Evaluation	
	**Types of results captured in each study^b^, n**			
		Clinical	2	
	Sample size, median (IQR; range)		429.5 (242.75-616.25; 56-803)	
	Length of follow-up (months), median (IQR; range)		16 (12-20; 8-24)	
**Pilot study (n=2)**				
	**Communication component feature type, n**			
		Primary feature	1	
		Supplemental feature	1	
	Stage of study^a^		Feasibility and piloting	
	**Types of results captured in each study^b^, n**			
		Usage	1	
		Usability	1	
		Clinical	1	
	Sample size, median (IQR; range)		370.5 (210.75-530.25; 51-690)	
	Length of follow-up (months), median (IQR; range)		6.25 (4.875-7.625; 3.5-9)	
**Website analysis (n=1)**				
	**Communication component feature type, n**			
		Primary feature	0	
		Supplemental feature	0	
		Not specified	1	
	Stage of study^a^		Evaluation	
	**Types of results captured in each study^b^, n**			
		Usage	1	
	Sample size, n		38	
	Length of follow-up (months), n		6	

^a^The definitions for the terms were derived from the 2008 Medical Research Council Framework for Evaluation of Complex Interventions.

^b^All results in the study have been included. The multiple outcomes are counted from the individual studies.

##### Study Outcomes

Table 4 presents the outcomes captured. A majority of the RCT studies (n=19 outcomes measured) focused on the clinical outcome, that is, a decrease in the blood pressure (8, 42%). Additionally, several studies investigated other outcomes such as behavioral changes (5, 26%), acceptability (2, 11%), cost-effectiveness (1, 5%), psychological effects (1, 5%), usage (1, 5%), and willingness (1, 5%). On the other hand, the nonrandomized studies (n=6 outcomes measured) investigated the clinical outcomes (3, 50%), usage (2, 33%), and usability (1, 17%) of the above web-based tools.

## Discussion

### Principal Findings

To our knowledge, this is the first study that has conducted a scoping review of published studies that used web-based tools for patients with hypertension. The researchers found 15 published studies that used web-based tools that could be helpful for patients with hypertension. These web-based tools were predominantly accessed from websites and mainly functioned as educational websites for diet, physical activity, or both. Few web-based tools provided multiple functions and features that enabled patient to access a patient portal, health records, or making appointments. Tools were used for behavior and lifestyle modifications, medication adherence, symptom reporting, and care planning.

Some of the tools enabled patients to communicate with health care providers such as physicians, nurses, and other allied health practitioners. Most tools were studied in the hypertension context, and some studies used them to evaluate multiple types of diseases including cardiovascular disease, diabetes, metabolic syndrome, hyperlipidemia, and obesity. More than half of the studies were RCTs that focused on clinical outcome evaluation, and several of them were observed behavioral changes. However, nonrandomized studies also examined impact on outcomes such as usage and usability.

### Characteristics of the Web-Based Tools

The results indicated that the studies used the web-based tools on their websites as the mode of communication in order to improve the health of the patients, as they offered a lot of health-related information and attempted to educate the patients. A few web-based tools were included on a multifunctional platform for providing electronic health records (2, 13%), a patient portal (1, 7%), and a digital management program (1, 7%).

Additionally, a majority of these tools were used by health care providers such as physicians (9, 60%), allied health professionals (5, 33%), nurses (5, 33%), or pharmacists (2, 13%). The researchers noted that a multifunctional platform facilitates the patients and the health care practitioners as they are both able to simultaneously track the health records, objectives, and plans. Furthermore, this platform could help them derive all information and educate the patients.

Many studies stated that information technology (IT)–based products could be very helpful in the health care sector (health IT) as they allowed practitioners to easily communicate with their patients. They could also report some improvement in medication adherence or appointment compliance among the patients. These studies further showed that the use of health IT tools helped the patients connect with their health care providers. This improved the general communication between both the parties, encouraged the patients to become more autonomous, and empowered the patients to manage their personal health [17-19]. Furthermore, health IT offers benefits to the patients as these tools allowed the providers to offer a personalized health care plan to their patients.

Digital personalized health plans are a new, patient-targeted health care system that can improve the conventional health care system. It is seen to be a cost-effective technique that increases the coordination of health care and engagement rate of the patients. This, in turn, improves the outcome [20].

However, technological knowledge is usually accompanied by many challenges like a lack of digital literacy [21]. Based on the selected studies, the researchers noted that a few patients did not consent to participate in these studies as they were not well-educated or were elderly and lacked a digital education [22]. A different study determined the reasons why the patients refused to participate in such studies and noted that these patients lacked computer skills, had no internet facilities, did not understand that the digital, web-based tools could offer any benefits, or lacked time, but still participated in the conventional cardiac rehabilitation programs [23]. Harris et al stated that the people who showed a low health literacy rate and experienced health discrepancies were similar to the people who had a limited or no access to internet facilities, digital devices or showed a low digital literacy [24].

Hence, it is necessary to offer additional training to people before providing them with a health IT intervention tool to prevent any unintended risk to the users [25]. Stellefson et al stated that skill-building activities need to be integrated into the comprehensive patient education programs to allow the patients, particularly those with a low education level or limited knowledge, to identify the high-quality sources that provided web-based health information that was related to their diseases [26].

In this study, the researchers noted that many of the web-based tools (13, 87%) encouraged a behavioral or lifestyle modification, while some others included additional features like medication adherence (6, 40%), health care planning (3, 20%), or symptom reporting (1, 7%). Most of these lifestyle modifications focused on diet, physical activities, alcohol or smoking behavior, and physical activities.

Many theories were used in the selected studies like the health belief model, theory of planned behavior, social cognitive theory, transtheoretical model of behavior change, and cognitive-behavioral approach [27-31]. A few of the health care providers were also trained in motivational interviews [31,32]. The researchers noted that the implementation of these theories while designing informational or educational websites could help in improving the outcome.

Liu et al defined some behavioral goals for the participants depending on their readiness, which increased the efficacy of initiating any change and reviewing the cognitive and social behavioral skills for preventing relapse and maintaining adherence [27]. This was further supported by a different study wherein the participants were guided through the different readiness stages so that they adhered to the self-care behavior [28].

In their study, Sabooteh et al implemented the various stages of the behavior theory in the software-based and web-based educational intervention programs for improving the physical activity levels of the students [33]. According to the researchers, if the design of the intervention program was based on the preparation level of the patients to change and use novel educational tools, it showed a higher positive effect on their progress levels during different stages of their behavioral change levels.

Moeini et al assessed the efficiency of web-based depression improvement programs that were based on social cognitive therapy [34]. They observed that these intervention programs were able to improve the depression levels of the participants. This supported the fact that application of theories while designing the website intervention programs could improve the final result.

Here, the researchers have noted that different techniques were used by earlier studies for educating people or offering vital information. This information could be provided with the help of online information manuals, weblinks, or chat features (involving both video and text). This could be accessed through the patient portal or sending messages and emails which contain some weblinks to the patients.

The International Usability Professionals’ Association stated that websites need to apply Section 508 to help every person, including those who face a difficulty hearing, seeing, or making accurate movements, so that they could use these links (US Department of Health and Human Services) [35]. Furthermore, the website content was an important aspect and must include very familiar words, while avoiding some terms that can confuse the users [35].

However, Lv et al held additional activities like cooking classes, healthy shopping tours, and educational webinars on the behavioral changes in order to improve patient engagement, rather than only distributing web-based educational handouts [29]. However, none of these engagement measures were related to the achievement of their blood pressure goals.

Similarly, Lussier et al compared the different approaches which included only the websites links and those that included web-based educational programs, followed by the workshop with an interactive nurse-facilitated small patient group [36]. They noted that the combined technique was not as effective in comparison to only the website-based approach, though they showed similar efficacy. This was attributed to the fact that the workshops were perceived as being outside of the routine care [37]. 

### Evaluation Characteristics

Most of the studies included in this review focused on RCTs. The data from these studies indicated that many of the researchers compared website interventions with traditional interventions. Some of these studies assessed different aspects of the outcomes, including the clinical results. A few of them also measured several outcomes like usability, psychological results, usage, cost-effectiveness, and willingness.

However, there was not a lot of evidence regarding the usability of a website with the clinical outcomes, which highlighted the need to carry out website interventions. This step supported the use of a website-based educational program in the existing intervention techniques to improve existing clinical practices. A majority of these studies were conducted over a long period (ie, more than 12 months) with a follow-up and included 264 participants on average. However, positive results were noted within 3-4 months of assessment. For example, in their study, Kao et al observed that the patients who were offered a website-based intervention program showed a remarkable improvement in their clinical outcomes within 3 months of their assessment compared to the control group of patients [38]. 

In this study, the researchers noted that most of the outcomes showed a positive result after 12 months rather than the initial 3-4 months. This was supported by another study carried out by Cernvall et al wherein the researchers assessed the long-term efficacy of the internet-based guided self-help program (with a 12-month follow-up schedule) for the parents of children undergoing chemotherapy [39]. Their study indicated that the use of internet tools that offered psychological intervention was a very effective medium of delivery.

Murray et al investigated a web-based self-management program that was used by people suffering from type 2 diabetes for improving their glycemic control and decreasing diabetes-related stress levels within 12 months [40]. A significant result was noted among the people using the program. Hence, the researchers stated that by offering web-based information to these patients, the health care providers could help them improve their overall health. This was regarded as an additional tool and could be used along with the existing self-management support techniques. This further showed that a website intervention program conducted for 12 months offered the best result.

### Article Characteristics

Most of the above-mentioned studies were carried out in North America, especially in the United States (5, 33%) and Canada (4, 27%). A few studies were carried out in countries like the United Kingdom, Korea, Spain, Italy, and Taiwan. Though a website-based intervention technique was an effective tool for providing vital information to the hypertensive patients, the health care budgets were attributed as the primary reason why a majority of these studies were conducted in North America (n=9).

The results of this study also indicated that the highest number of papers on this topic were published between 2011 and 2012. Thereafter, very few reports were published between 2013 and 2015. This publication trend gradually increased between 2016 and 2019. One of the first reports that were published on this topic was carried out in the United States in 2011, followed by 2 papers that were published from Korea in 2012. Over the years, many studies that assessed the various website-based intervention tools that could be used by hypertensive patients were published from other countries.

Based on these observations, website-based education is an effective educational tool since it can be flexible and used by the patients anytime and anywhere. Laine et al noted that these website-based tools were very helpful as they offered the patients very vital information [41]. Furthermore, these tools also improved the mental state of the patients since they could control their physical health [42]. Thus, the patients could increase their knowledge and autonomously use and improve their health status.

### Study Outcomes

The web-based tools were helpful for patients with hypertension. The selected studies showed positive results, which indicated that the patients with hypertension showed a significant improvement in their health when they incorporated the website-based tools into their regular schedule. Some of the studies measured the clinical outcomes of using these tools. One of the best clinical outcomes that were noted after the use of these tools was the marked decrease in the systolic and diastolic blood pressure of the patients. These were the main indicators that helped in assessing the “performance” of the website-based tools [43].

Here, the researchers have also noted that there was a significant decrease in the systolic blood pressure levels of the patients who used these tools for 12 months. Similar results were noted when traditional counselling was offered to the patients. Thus, it was concluded that the application of a web-based intervention tool in the clinical practices could improve the hypertension levels of the patients as these tools taught the patients to control their health status. Furthermore, the implementation of this technique encouraged them to execute long-term lifestyle changes and thus decrease their blood pressure levels [28]. 

Some of the selected studies also used usage and willingness, of the patients and the tools’ acceptability to the patients as important outcomes while measuring the effective nature of the website-based tools. They concluded that many people were willing to take part in a web-based educational program, based on the number of website visitors and returning visitors. By assessing these outcomes, the researchers determined the acceptability and the suitability of their website and found that it was important to effectively plan and then design a high-quality education website. It must include many multimedia elements, a higher accessibility level, and many interactive features [44].

Due to the advancement in the field of IT and the availability of online health information, website-based education could help in determining the health care behavior of patients and disease management [42]. Hence, the researchers in this study have proposed the need to determine the preferences of patients with regard to the features and design of educational websites for patients with hypertension. They aim to use patient-tailored information and design a website which offers interactivity, clear content presentation, content credibility, multimedia content and interpretability of the content, and other helpful features for educating the patients regarding their hypertension management.

However, this study displayed some limitations. The researchers used a scoping review technique, which identified the published articles at the expense of carrying out an in-depth analysis of the studies. The researchers aimed to carry out a comprehensive analysis using an extensive keyword search and search string for each database; however, they could have missed a few important studies, since there is a lack of standard terminologies that could be used in this area. The researchers also limited their review to include only studies published in English or translated in the English language. They could have missed some studies that were not translated to the English language or were not accessible from the databases used in the study, since many studies are published in countries that do not use English as their primary language.

### Conclusions

Hypertension, or high blood pressure, is a trigger for many chronic diseases like cardiovascular diseases, diabetes, kidney failure, and strokes. Patients with hypertension can derive many benefits after using a web-based dietary and physical activity intervention program, which could help them make many lifestyle changes.

Here, the researchers selected 15 published studies that offered website-based intervention programs that included several interactive features for decreasing the blood pressure levels of the patients. These websites differed based on their content, usability, accessibility, and delivery method (eg, text, video, or audio). Before selecting a website program for their use, the patients considered many factors like ease of use, accessibility, presence of additional features, and whether the offered information was supported by some evidence.

Hence, there is a lot of scope for improving some issues, such as the need for websites to be tested for their usability, acceptability, and resulting clinical outcomes. Based on these factors, it was determined whether the website could be implemented to decrease the blood pressure levels of the patients in a clinical setting. Use of these websites could decrease health care costs and the number of appointments since many people can access these websites at the same time, from different regions. Additionally, the efficiency and the quality of the care that is provided could be maintained or even improved.

## Appendix

Multimedia Appendix 1 Search string.

## Abbreviations

IT: information technology
LMICs: low- and middle-income countries
PRISMA-ScR: Preferred Reporting Items for Systematic reviews and Meta-Analyses extension for Scoping Reviews
RCT: randomized controlled trial
