# Supplementary material for: Web-Based Dietary and Physical Activity Intervention Programs for Patients With Hypertension: Scoping Review
Source: J Med Internet Res. 2021 Mar 15;23(3):e22465. doi: 10.2196/22465 (PMC8074856; doi:10.2196/22465)
Supplement: Multimedia Appendix 1 [file jmir_v23i3e22465_app1.docx]

**Multimedia Appendix 1. Search string.**

| No. | Search Database Used | Search String | Search Term |
| --- | --- | --- | --- |
| 1. | Google Scholar | allintitle: ("web based”) AND ("hypertension" OR "high blood = pressure" OR "hypertensive" ) AND (“patient”) | Web-based |
| 2. | Google Scholar | allintitle: ("mobile health”) AND ( "hypertension" OR "high blood pressure" OR "hypertensive" ) AND (“patient”) | mobile health |
| 3. | Google Scholar | allintitle: ("internet”) AND ( "hypertension" OR "high blood pressure" OR "hypertensive" ) AND (“patient”) | internet |
| 4. | Google Scholar | allintitle: ("digital”) AND ( "hypertension" OR "high blood pressure" OR "hypertensive" ) AND (“patient”) | digital |
| 5. | Google Scholar | allintitle: ("e-health”) AND ( "hypertension" OR "high blood pressure" OR "hypertensive" ) AND (“patient”) | e-health |
| 6. | Google Scholar | allintitle: ("internet-enabled interactive multimedia”) AND ( "hypertension" OR "high blood pressure" OR "hypertensive" ) AND (“patient”) | internet-enabled interactive multimedia |
| 7. | Google Scholar | allintitle: ("technology-assisted”) AND ("hypertension" OR "high blood pressure" OR "hypertensive" ) AND (“patient”) | technology-assisted |
| 8. | Google Scholar | allintitle: ("internet-supported”) AND ("hypertension" OR "high blood pressure" OR "hypertensive" ) AND (“patient”) | internet-supported |
| 9. | Google Scholar | allintitle: ("online”) AND ("hypertension" OR "high blood pressure" OR "hypertensive" ) AND (“patient”) | online |
| 10. | Google Scholar | allintitle: ("computer-assisted”) AND ("hypertension" OR "high blood pressure" OR "hypertensive" ) AND (“patient”) | computer-assisted |
| 11. | Google Scholar | allintitle: ("interactive computer-based”) AND ("hypertension" OR "high blood pressure" OR "hypertensive" ) AND (“patient”) | interactive computer-based |
| 12. | Google Scholar | allintitle: ("computer-based”) AND ("hypertension" OR "high blood pressure" OR "hypertensive" ) AND (“patient”) | computer-based |
| 13. | Google Scholar | allintitle: ("website-delivered”) AND ("hypertension" OR "high blood pressure" OR "hypertensive" ) AND (“patient”) | website-delivered |
| 14. | Google Scholar | allintitle: ("e-learning”) AND ("hypertension" OR "high blood pressure" OR "hypertensive" ) AND (“patient”) | e-learning |
| 15. | Google Scholar | allintitle: ("computer-delivered”) AND ("hypertension" OR "high blood pressure" OR "hypertensive" ) AND (“patient”) | computer-delivered |
| 16. | Google Scholar | allintitle: ("internet-based”) AND ("hypertension" OR "high blood pressure" OR "hypertensive" ) AND (“patient”) | internet-based |
| 17. | Google Scholar | allintitle: ("website”) AND ("hypertension" OR "high blood pressure" OR "hypertensive" ) AND (“patient”) | website |
| 18. | Google Scholar | allintitle: ("digital health technology”) AND ("hypertension" OR "high blood pressure" OR "hypertensive" ) AND (“patient”) | digital health technology |
| 19. | Google Scholar | allintitle: ("digital medicines”) AND ("hypertension" OR "high blood pressure" OR "hypertensive" ) AND (“patient”) | digital medicines |
| 20. | Google Scholar | allintitle: ("web application”) AND ( "hypertension" OR "high blood pressure" OR "hypertensive" ) AND (“patient”) | web application |
| 21. | Pubmed | (((web based) AND (nutrition* intervention OR diet* intervention OR physical activity OR exercise)) AND (hypertension OR high blood pressure OR hypertensive)) AND patients | web based |
| 22. | Pubmed | ((((internet OR mobile health OR digital or e-health)) AND (nutrition* intervention OR diet* intervention OR physical activity OR exercise)) AND (hypertension OR high blood pressure OR hypertensive)) AND patients | internet/mobile health/digital/e-health |
| 23. | Pubmed | ((((internet-enabled interactive multimedia OR technology-assisted OR internet-supported OR online)) AND (nutrition* intervention OR diet* intervention OR physical activity OR exercise)) AND (hypertension OR high blood pressure OR hypertensive)) AND patients | internet-enabled interactive multimedia/technology-assisted/internet-supported/online |
| 24. | Pubmed | ((((computer-assisted OR interactive computer-based OR computer-based OR website-delivered OR e-learning OR computer-delivered)) AND (nutrition* intervention OR diet* intervention OR physical activity OR exercise)) AND (hypertension OR high blood pressure OR hypertensive)) AND patients | computer-assisted/interactive computer-based/computer-based/website-delivered/e-learning/computer-delivered |
| 25. | Pubmed | ((((internet-based OR website OR digital health technology OR digital medicines)) AND (nutrition* intervention OR diet* intervention OR physical activity OR exercise)) AND (hypertension OR high blood pressure OR hypertensive)) AND patients | internet-based /website /digital health technology /digital medicines |
| 26. | Pubmed | (((web application) AND (nutrition* intervention OR diet* intervention OR physical activity OR exercise)) AND (hypertension OR high blood pressure OR hypertensive)) AND patients | web application |
| 27. | Scopus | TITLE-ABS-KEY ( ( "web based" OR "mobile health" OR "internet" OR "digital" OR "e-Health" OR "internet-enabled interactive multimedia" OR "technology-assisted" OR "internet-supported" OR "online" OR "computer-assisted" OR "interactive computer-based" OR "computer-based" OR "website-delivered" OR "e-learning" OR "computer-delivered" OR "internet-based" OR "website" OR "digital health technology" OR "digital medicines" ) AND ( "diet* intervention" OR "nutrition* intervention" OR "physical activity" OR "exercise" ) AND ( "hypertension" OR "high blood pressure" OR "hypertensive" ) AND ( "patients" ) ) AND ( LIMIT-TO ( PUBYEAR , 2020 ) OR LIMIT-TO ( PUBYEAR , 2019 ) OR LIMIT-TO ( PUBYEAR , 2018 ) OR LIMIT-TO ( PUBYEAR , 2017 ) OR LIMIT-TO ( PUBYEAR , 2016 ) OR LIMIT-TO ( PUBYEAR , 2015 ) OR LIMIT-TO ( PUBYEAR , 2014 ) OR LIMIT-TO ( PUBYEAR , 2013 ) OR LIMIT-TO ( PUBYEAR , 2012 ) OR LIMIT-TO ( PUBYEAR , 2011 ) OR LIMIT-TO ( PUBYEAR , 2010 ) ) AND ( LIMIT-TO ( DOCTYPE , "ar" ) ) AND ( LIMIT-TO ( LANGUAGE , "English" ) ) |  |
| 28. | Scopus | TITLE-ABS-KEY ( ( "web application" ) AND ( "diet* intervention" OR "nutrition* intervention" OR "physical activity" OR "exercise" ) AND ( "hypertension" OR "high blood pressure" OR "hypertensive" ) AND ( "patients" ) ) AND ( LIMIT-TO ( DOCTYPE , "ar" ) ) AND ( LIMIT-TO ( PUBYEAR , 2020 ) OR LIMIT-TO ( PUBYEAR , 2019 ) OR LIMIT-TO ( PUBYEAR , 2018 ) OR LIMIT-TO ( PUBYEAR , 2017 ) OR LIMIT-TO ( PUBYEAR , 2016 ) OR LIMIT-TO ( PUBYEAR , 2015 ) OR LIMIT-TO ( PUBYEAR , 2014 ) OR LIMIT-TO ( PUBYEAR , 2013 ) OR LIMIT-TO ( PUBYEAR , 2012 ) OR LIMIT-TO ( PUBYEAR , 2011 ) OR LIMIT-TO ( PUBYEAR , 2010 ) ) AND ( LIMIT-TO ( LANGUAGE , "English" ) ) | web application |
| 29. | WOS | #1 (TS=(web based OR internet OR mobile health OR digital OR e-health OR internet-enabled interactive multimedia OR technology-assisted OR internet-supported OR online OR computer-assisted OR interactive computer-based OR computer-based OR website-delivered OR e-learning OR computer-delivered OR internet-based OR website OR digital health technology OR digital medicines)) AND LANGUAGE: (English) AND DOCUMENT TYPES: (Article) Indexes=SCI-EXPANDED, SSCI, A&HCI, CPCI-S, CPCI-SSH, BKCI-S, BKCI-SSH, ESCI Timespan=2010-2020 |  |
|  |  | #2 (TS=(diet* intervention OR nutrition* intervention OR physical activity OR exercise)) AND LANGUAGE: (English) AND DOCUMENT TYPES: (Article) |  |
|  |  | #3 (TS=(hypertension OR high blood pressure OR hypertensive)) AND LANGUAGE: (English) AND DOCUMENT TYPES: (Article) |  |
|  |  | #4 (TS=(patients)) AND LANGUAGE: (English) AND DOCUMENT TYPES: (Article) |  |
|  |  | #4 AND #3 AND #2 AND #1 |  |
| 30. | WOS | #1 (TS=(web application)) AND LANGUAGE: (English) AND DOCUMENT TYPES: (Article) Indexes=SCI-EXPANDED, SSCI, A&HCI, CPCI-S, CPCI-SSH, BKCI-S, BKCI-SSH, ESCI Timespan=2010-2020 | web application |
|  |  | #2 (TS=(diet* intervention OR nutrition* intervention OR physical activity OR exercise)) AND LANGUAGE: (English) AND DOCUMENT TYPES: (Article) |  |
|  |  | #3 (TS=(hypertension OR high blood pressure OR hypertensive)) AND LANGUAGE: (English) AND DOCUMENT TYPES: (Article) |  |
|  |  | #4 (TS=(patients)) AND LANGUAGE: (English) AND DOCUMENT TYPES: (Article) |  |
|  |  | #4 AND #3 AND #2 AND #1 |  |
| 31. | Science Direct | (("web based" OR "web application") AND ("nutrition* intervention" OR "diet* intervention" OR "physical activity" OR "exercise") AND ( "hypertension" OR "high blood pressure" OR "hypertensive") AND ("patients")) | web based/ web application |
| 32. | Science Direct | (("mobile health" OR "internet" OR "digital") AND ("nutrition* intervention" OR "diet* intervention" OR "physical activity" OR "exercise") AND ( "hypertension" OR "high blood pressure" OR "hypertensive") AND ("patients")) | mobile health/ internet/ digital |
| 33. | Science Direct | (("e-health" OR "internet-enabled interactive multimedia") AND ("nutrition* intervention" OR "diet* intervention" OR "physical activity" OR "exercise") AND ( "hypertension" OR "high blood pressure" OR "hypertensive") AND ("patients")) | e-health/internet-enabled interactive multimedia |
| 34. | Science Direct | (("technology-assisted" OR "internet-supported" OR "online") AND ("nutrition* intervention" OR "diet* intervention" OR "physical activity" OR "exercise") AND ( "hypertension" OR "high blood pressure" OR "hypertensive") AND ("patients")) | technology-assisted/internet-supported/online |
| 35. | Science Direct | (("computer-assisted" OR "interactive computer-based" OR "e-learning") AND ("nutrition* intervention" OR "diet* intervention" OR "physical activity" OR "exercise") AND ( "hypertension" OR "high blood pressure" OR "hypertensive") AND ("patients")) | computer-assisted/interactive computer-based/e-learning |
| 36. | Science Direct | (("computer-based" OR "website-delivered" OR "computer-delivered") AND ("nutrition* intervention" OR "diet* intervention" OR "physical activity" OR "exercise") AND ( "hypertension" OR "high blood pressure" OR "hypertensive") AND ("patients")) | computer-based/website-delivered/computer-delivered |
| 37. | Science Direct | (("internet-based" OR "website" OR "digital health technology") AND ("nutrition* intervention" OR "diet* intervention" OR "physical activity" OR "exercise") AND ( "hypertension" OR "high blood pressure" OR "hypertensive") AND ("patients")) | internet-based/website/digital health technology |
| 38. | Science Direct | (("digital medicines") AND ("nutrition* intervention" OR "diet* intervention" OR "physical activity" OR "exercise") AND ( "hypertension" OR "high blood pressure" OR "hypertensive") AND ("patients")) | digital medicines |
| 39. | EBSCOhost: MEDLINE | AB (web based OR mobile health) AND AB (diet* intervention OR nutrition* intervention OR physical activity OR exercise) AND (hypertension OR hypertensive OR high blood pressure) AND patient | web based/ mobile health |
| 40. | EBSCOhost: MEDLINE | AB (internet OR digital OR e-health) AND AB (diet* intervention OR nutrition* intervention OR physical activity OR exercise) AND (hypertension OR hypertensive OR high blood pressure) AND patient | internet/digital/e-health |
| 41. | EBSCOhost: MEDLINE | AB (internet-enabled interactive multimedia OR technology-assisted OR internet-supported OR online) AND AB (diet* intervention OR nutrition* intervention OR physical activity OR exercise) AND (hypertension OR hypertensive OR high blood pressure) AND patient | internet-enabled interactive multimedia/technology-assisted /internet-supported/ online |
| 42. | EBSCOhost: MEDLINE | AB (computer-assisted OR interactive computer-based OR computer-based OR website-delivered) AND AB (diet* intervention OR nutrition* intervention OR physical activity OR exercise) AND (hypertension OR hypertensive OR high blood pressure) AND patient | computer-assisted/interactive computer-based/computer-based/website-delivered |
| 43. | EBSCOhost: MEDLINE | AB (e-learning OR computer-delivered OR internet-based OR website) AND AB (diet* intervention OR nutrition* intervention OR physical activity OR exercise) AND (hypertension OR hypertensive OR high blood pressure) AND patient | e-learning/computer-delivered/internet-based/website |
| 44. | EBSCOhost: MEDLINE | AB (digital health technology OR digital medicines OR web application) AND AB (diet* intervention OR nutrition* intervention OR physical activity OR exercise) AND (hypertension OR hypertensive OR high blood pressure) AND patient | digital health technology/ digital medicines /web application |
